# Augmenting the Disaster Healthcare Workforce

**DOI:** 10.5811/westjem.2020.4.47553

**Published:** 2020-04-13

**Authors:** Kenneth V. Iserson

**Affiliations:** University of Arizona, Department of Emergency Medicine, Tucson, Arizona

## Abstract

In disasters such as the COVID-19 pandemic, we need to use all available resources to bolster our healthcare workforce. Many factors go into this process, including selecting the groups of professionals we will need, streamlining their licensing and credentialing processes, identifying appropriate roles for them, and supporting their health and well-being. The questions we must answer are these: How many staff will we need? How do we provide them with emergency licenses and credentials to practice? What interstate licensing compacts and registration systems exist to facilitate the process? What caveats are there to using retired healthcare professionals and healthcare students? How can we best avoid attrition among and increase the numbers of international medical graduates? Which non-clinical volunteers can we use and in what capacities? The answers to these questions will change as the crisis develops, although the earlier we address them, the smoother will be the process of using augmentees for the healthcare system.

## INTRODUCTION

### How many staff will we need?

At the onset of a disaster, or when many unknowns exist, it is not clear how many healthcare staff will be needed. Healthcare administrators and emergency management must attempt to calculate needs based on projected illnesses and existing capacities. Based on a 40% prevalence of COVID-19 in the United States during the pandemic, 100 million people infected, about 21 million will be hospitalized, with about 4.5 million of them needing intensive care. While healthcare systems across the country have varying abilities to accommodate this patient load, a six-month epidemic will result in filling 275% of the potentially available capacity of inpatient beds and >500% of the intensive care unit (ICU) bed capacity. If the course is flattened to 12 months, the need for hospital beds would be 137% and the ICU beds 254% of capacity.^1^ Either situation will require a significant increase in our physician, nurse, and associated workforce.

Increased bed capacity will also necessitate additional doctors and nurses. As of late March, New York was increasing existing hospitals’ bed capacity by at least 50%, doubling the number of ICU beds, and waiting for the US Army to convert four sites into temporary hospitals for people needing less intensive care or who are recovering.^2^ Washington State has received 1000 hospital beds from the military, and California, anticipating a need for more than 19,000 new beds, is opening multiple temporary hospitals, including reopening some that had closed. Florida is similarly opening multiple hospitals with thousands of beds.^2^

In disaster situations, although most healthcare professionals will want to respond, whether they actually do so, and then continue to do so, depends on the risk they perceive to themselves and their families, the measures being taken to keep them safe, the value they see in their work, the completeness and transparency of the information they are given about the developing situation, and their personal (professional, religious, and other) values.^3^,^4^

Once healthcare professionals decide to stay, their numbers will inevitably decrease due to the vast influx of patients and the expanded roles they must perform, leading to burnout, as well as to illness-related attrition, especially in epidemics. For example, 14% of Spain’s first 40,000 confirmed coronavirus cases were medical professionals. Catalonia’s Igualada Hospital put 30% of its staff in home isolation. Similar scenarios have occurred across Europe.^5^ The above examples demonstrate that additional physicians, nurses, and support staff will be needed. Most will be volunteers. Such volunteers can augment regular staff if careful thought is given to the vetting process, the roles they are assigned, and the support they will receive.

### Which healthcare professionals are needed?

A wide variety of healthcare professionals can help augment normal staff (Table 1). Many of them will be asked to expand their scope of practice. As the American College of Chest Physicians wrote, “During a disaster, all work usually performed may not be ‘essential’ but all health-care workers are essential. The goal . . . is to match the caregiver competencies with patient needs. To that end . . . the scope of practice and experience of various caregivers, should be used to assign caregivers to the patients.”^6^

Primary clinicians who may have normally worked in a variety of specialties can be assigned to positions in which they will be useful. All physicians, advanced nurse practitioners, and physician assistants can help in their areas of expertise or in fast-track and primary care, permitting the full-time staff to treat the sickest patients. Surgeons of all types, podiatrists, and some dentists (eg, oral surgeons) can assist in the emergency department (ED), clinics, and the operating room. Volunteer healthcare professionals can also staff alternative care sites. These extensions of clinicians’ scopes of practice are informal; they are based on supervisors’ knowledge of an individual’s capabilities and on extenuating circumstances. When a practitioner is inexperienced, he or she is supervised by a current staff member.

In the areas hardest hit by the pandemic, nurses, especially those with ED and critical care experience, are in short supply.^7^ While experienced nurses may be asked to diagnose and prescribe treatments during disasters, some states have curtailed some advanced practice nurses’ work. Although these nurses routinely assume primary care responsibilities, 12 states are still restricting them to practice only with a supervising physician.^8^ Pennsylvania, in contrast, has changed its rules to allow family care nurse practitioners (and retail pharmacists) to care for COVID-19 patients, if needed.^9^

Certified but unlicensed paramedics and emergency medical technicians can perform a variety of healthcare services, depending on their training and experience. Using alternative care practitioners may be beneficial for some populations; each facility must decide for itself which category to use.

## EMERGENCY LICENSING AND CREDENTIALING OF HEALTHCARE PROFESSIONALS

There are two issues to confront when using healthcare professionals to augment the system: state licensing, and institution-specific credentials to perform diagnostic and therapeutic procedures. If healthcare professionals volunteer within a state in which they are licensed, hospitals need only credential them to perform clinical duties. This process becomes a bit more flexible than normal during disasters. Permitting out-of-state healthcare professionals to work requires not only verifying their *licenses*, but also getting the state to grant specific permission; many states are now doing so under the Uniform Emergency Volunteer Health Practitioner Act (UEVHPA) or a separate emergency exception.

As of 2020, 18 states and the District of Columbia have enacted UEVHPA, which allows them to recognize out-of-state licenses for a variety of health practitioners during a state of declared emergency. Other states, such as New York, Florida, South Carolina, Georgia, and Texas, have separately loosened licensure requirements for physicians, nurses, advanced nurse practitioners, or pharmacists. Current state-specific information about licensure requirements during the COVID-19 epidemic is available through the National Conference of State Legislatures (www.ncsl.org/research/labor-and-employment/covid-19-occupational-licensing-in-public-emergencies.aspx).^10^ To make it easier for hospitals and healthcare agencies to verify physicians and physician assistants’ licensing information, the Federation of State Medical Boards (FSMB) is offering them free access to its Physician Data Center (PDC), which contains licensure and disciplinary information for the more than one million physicians and physician assistants in the United States (US).^11^

Once individual licenses are verified, institutions must then ascertain which *credentials* to allow based on an individual’s skills and proficiency. The PDC helps hospitals to quickly verify physicians and physician assistants’ medical schools, training, any disciplinary actions, and specialty certifications.^12^ With this information, the Joint Commission permits hospitals to provide emergency credentialing/disaster privileges on an individual basis. For physicians, the process involves, at a minimum, presentation of a medical license and photo identification, and, usually, personal and malpractice coverage information.^13^ If the process is already contained in their medical staff bylaws, hospitals can then grant temporary privileges “to fulfill an important patient care, treatment, and service need,” such as that “the patient care volume exceeds the level that can be handled by currently privileged practitioners and additional practitioners are needed to handle the volume.”^14^ The hospital CEO normally grants the privileges after receiving the medical staff president’s recommendation.^14^

## ORGANIZED TEAMS

In the US, organized medical teams include the Disaster Medical Assistance Teams (DMAT) and the Public Health Service Commissioned Corps. In major disasters (eg, Hurricane Katrina, COVID-19), the military may also assist. The benefits of organized groups are that they are usually self-sufficient, have experience in disaster situations, and have the necessary personnel, including credentialed healthcare professionals. Health professionals on these teams may, by federal law, practice in any state where they have been deployed during disasters. They have also been pre-credentialed for specific diagnostic and therapeutic procedures. Unfortunately, one cannot rely on these organized disaster teams to respond immediately, especially if they must mobilize and travel considerable distances. Bureaucratic red tape often prevents them from arriving for a considerable time after a state requests them. In some cases, it has taken at least 10 days until they were on site and operational.

## INTERSTATE LICENSING COMPACTS

Several programs exist within the US to license and evaluate healthcare professionals’ general credentials so that they can work in other locations, either within their state or in other states during disasters. These are the Emergency Management Assistance Compact (EMAC), the Nurse Licensure Compact (NLC), and the Recognition of EMS Personnel Licensure Interstate CompAct (REPLICA).

The EMAC is an interstate mutual aid agreement to train for and to respond to emergency events, including natural and man-made disasters. Under EMAC, once a governor declares a disaster, the state can request assistance through the EMAC Operating System. A management team is sent to help the requesting state’s emergency operations center evaluate and obtain the appropriate resources. When EMAC has been activated, the receiving state recognizes responders’ licenses, certificates, and permits, “subject to such limitations and conditions as the governor of the requesting state may prescribe.”^15^ EMAC was ratified by the US Congress in 1996^16^ and has been adopted by all states, the District of Columbia, the US Virgin Islands, Puerto Rico, and Guam. The requesting state is responsible for reimbursing the assisting state for any expenses incurred. EMAC also addresses liability and compensation (sometimes through the Federal Emergency Management Agency).^17^–^19^

The NLC is an agreement between 34 states allowing nurses residing in and having a license in an NLC state to practice in other states that are part of the agreement. In 2018, the Enhanced Nursing Licensure Compact (eNLC) was implemented, requiring applicants to undergo state and federal, fingerprint-based, criminal background checks. Nurses working under the NLC must practice in accordance with the laws of the state where the patient is located and are subject to that state’s jurisdiction, licensing board, courts, and laws.^15^ Unfortunately, four of the states hardest hit thus far^20^ by COVID-19 (Washington, New York, Illinois, and California) are not signatories to this compact.^21^

REPLICA allows EMS personnel to work across state boundaries in the performance of their assigned EMS duties. It functions in both routine and disaster settings and has been approved by 27 states.^22^ REPLICA grants legal recognition to EMS personnel licensed in any other member state. Under the agreement, EMS personnel must complete an FBI biometric criminal background check when applying for a new EMS license and states must share licensing and disciplinary actions with the other participating states.^23^

A legal framework for the International Emergency Management Assistance Compact (IEMAC) already exists in Connecticut statutes. It describes a mutual aid system among six New England states and five Canadian provinces. However, no other jurisdiction has approved or implemented it. Its structure would provide licensure reciprocity for health professionals and other disaster workers aiding a government-initiated emergency response.^15^,^24^

## EMERGENCY SYSTEM FOR ADVANCE REGISTRATION OF VOLUNTEER HEALTH PROFESSIONALS CREDENTIALING SYSTEM (ESAR-VHP)

The ESAR-VHP is a federal program that established state registries for licensed and credentialed volunteer health professionals. Each state has its own registry process, generally requiring registrants to submit extensive documentation in advance of a crisis. Once a state’s governor declares an emergency and mobilizes the state emergency management office, hospitals and other healthcare facilities can use ESAR-VHP to obtain previously verified information about volunteers’ licenses, credentials, and accreditations, as well as training skills, competencies, and employment.^25^ Table 1 lists the healthcare professionals that states most commonly register.

The ESAR-VHP assigns volunteers into one of four credential levels, based on the verified documents they provide.

- Level 1: Clinically active in a hospital, either as an employee or by having hospital privileges.
- Level 2: Clinically active in a wide variety of settings (eg, clinics, nursing homes, and shelters).
- Level 3: Meets the basic qualifications necessary to practice.
- Level 4: Those with healthcare experience or education that would be useful when assisting clinicians and providing basic healthcare not controlled by the scope of practice laws (eg, health professions students or retired health professionals who no longer hold a license).^26^

Many ESAR-VHP registrants participate through the Medical Reserve Corps (MRC), a national network of 175,000 volunteers in about 850 locally organized and activated units. Despite its name, MRC volunteers include not only medical and public health professionals, but also community members without healthcare backgrounds.^27^ As of late March 2020, approximately 100 MRC units were supporting COVID-19 response activities, especially assisting with call center operations (eg, fielding inquiries from the general public and local medical providers), community education, patient case and contact investigations, and patient monitoring. Some have been asked to support patient testing efforts and surge staffing needs (eg, hospitals, alternate care sites, and EMS).^25^

## RETIREES, STUDENT VOLUNTEERS AND INTERNATIONAL MEDICAL GRADUATES

Many volunteers in a disaster setting will be retired clinicians or healthcare students. Both can be problematic for different reasons. International medical graduates (IMG) present a host of benefits and problems.

### Retirees

In mid-March, Spain began the emergency recruitment of 50,000 healthcare workers, ranging from medical students to retired doctors.^28^ In what the United Kingdom’s medical director called “outbreaks of altruism,” thousands of retired physicians and nurses are returning to work in the National Health Service (NHS) at the government’s request.^29^

According to data from the Federation of State Medical Boards, most US states “are loosening their licensing rules to give those with clinical skill the ability to pitch in, such as allowing out-of-state physicians to practice right away, asking retired physicians to volunteer, and more.” 30 In late March, New York’s Health Commissioner said the state would welcome retirees and those with expired licenses to return to clinical medicine.^2^ Pennsylvania is permitting physicians who retired within the past five years to reactivate their medical licenses at no cost. It also waived licensing requirements for both in-state and out-of-state healthcare providers to treat patients via telemedicine.^9^ As it begins to augment overwhelmed civilian medical systems, the US Army has solicited its retired doctors, nurses, and medics who have served in critical care or EMS positions to return to work if they are not already employed in the civilian sector.^31^

New York State officials have said that they will recertify individuals for immediate deployment.^2^ Those returning to work in the NHS after >3 years’ retirement, however, will take a short refresher course. This highlights one problem with this group of (usually) older individuals. Their knowledge of current practice may be outdated. In some cases, such as in the COVID-19 pandemic, they also may be at much higher risk of dying, although once an immunoglobulin G test is readily available, those found to have had the disease may be immune.^32^ Many of them also may have other health-related problems.^30^ This requires individual screening by a knowledgeable professional. In addition, they will need malpractice insurance, which they undoubtedly lack. On the other hand, they will bring a lifetime of knowledge and experience, and often amazing skill in those procedures with which they are familiar.

### Students

Healthcare student volunteers pose the opposite problem. Their health and cognitive abilities are rarely an issue. Yet, depending on their level of training, they may have insufficient experience to be useful in many critical areas. More importantly, it raises the ethical issue of whether society should potentially sacrifice many next-generation practitioners to function in a potentially minor role. While in the UK more than 24,000 final-year student nurses and doctors are starting to work in the NHS,^29^ US healthcare students have mostly been pulled off clinical rotations.^33^ New York University Medical School offered its senior medical students who meet all graduation and screening requirements the voluntary opportunity to graduate three months early so they can help relieve the strain on frontline clinicians. The plan is to have them immediately begin working as paid interns in their hospital’s internal medicine and emergency departments, although they do not have to continue in those specialties.^34^ Medical schools across the country have followed their lead. Many health professions students are assisting their colleagues in non-clinical ways, including staffing community and clinic information lines, helping to procure additional personal protective equipment, babysitting, grocery shopping, and doing other necessary chores for hospital workers, and volunteering on time-sensitive COVID-19 laboratory projects.^30^,^35^

### International Medical Graduates

So far, this group of generally well-trained additional physicians has received little attention. IMGs include those in training whose visas will soon expire, those accepted into US residency programs for July 1 but who cannot gain entry to the US; and those still unlicensed due to the requirement that they need to repeat a residency in the US. Dr. Irwin Redlener, director of the National Center for Disaster Preparedness at Columbia University, advocates revisiting the rules about internationally trained physicians who are living in the US. He believes that we should eliminate “for now—the regulation that you have to repeat your residency in order to practice in the US. These people are ready to go, and my experience with them is they’re very talented, very well trained and coming from all different countries. That’s a pool we should tap.”^30^

To address the dilemma of IMG physicians who may soon be compelled to leave the workforce against their will or who were supposed to begin residencies, the American Medical Association “urged immigration authorities to extend visas for foreign national physicians lawfully practicing in the US and for the Departments of State and Homeland Security to expedite visa processing to ensure that non-US citizen IMGs can enter the country to begin their residency training programs on July 1.”^36^ As of early April, the government has not taken any action to retain or acquire these physicians.

## NON-CLINICAL VOLUNTEERS

Many vital jobs in healthcare can be accomplished by those with no significant or non-healthcare experience (Table 2). In Britain, more than 500,000 volunteers are helping the NHS in non-clinical roles, primarily delivering food and medicines, driving patients to appointments, and phoning people with underlying health conditions who are isolating themselves from the virus by staying at home.^29^ The local and national Red Cross can supply a general workforce, but normally does not provide professional health care.

## SUPPORTING THE AUGMENTEES

Disaster work is stressful. Supervisors must carefully manage augmentees who may not be familiar with the facility, its procedures, and personnel, even if they are healthcare professionals. That includes carefully planning the group’s work schedules, housing, meals, and security. Burnout is common among disaster workers. Often this is due to workers’ unwillingness to step aside to let others assume their tasks at the end of a shift. Augmentees housed in the patient treatment facility may be unable to rest adequately, especially when sleeping and resting areas are not sufficiently isolated from patient care areas. Food keeps the army of disaster healthcare workers on its feet and helps maintain esprit de corps. With limited outside sources of prepared food coupled with long work hours, facilities should expect to feed staff 24 hours a day.^37^

The most important principle is to make safety the first consideration—in all situations. A necessary part of the overall security system is to supply some type of identification—preferably in a format that cannot be easily copied—to those volunteers who have authorization to enter the healthcare facility. For example, some nurses in New York City hospitals are benefiting from newly instated protections, including “nurses now being driven to and from work in private cars whose drivers are certified healthy, sealed lunches being delivered to their hospitals, childcare, and grocery deliveries to their families at home.”^7^ New York nurse Katherine Ramos, currently working at an ED and caring for two ill family members, said grocery delivery “made a major difference in her ability to continue to care for others and help flatten the curve. ‘They have not just been keeping me safe, but they’ve been keeping the rest of the populations safe, which is huge,’ Ramos said. ‘I don’t want to be the one spreading anything to anybody.’”^7^

Personal welfare is often disregarded in times of crisis. Experienced disaster team members suggest that encouraging augmentees to care for themselves will allow them to function better and longer. Specifically, ask them to rest whenever possible, hydrate frequently, eat often but generally avoid simple sugars and caffeine, and get exercise.^38^

## CONCLUSION

In disasters such as the COVID-19 pandemic, we need to use all available resources to bolster our healthcare workforce. Many factors go into this process, including selecting the groups of professionals we will need, streamlining their licensing and credentialing processes, identifying appropriate roles for them, and supporting their health and well-being.

## Figures and Tables

**Table 1:** Post-disaster healthcare volunteer categories.

Healthcare professionals (licensed)		
Physician	Dentist/Oral surgeon	Veterinarian
Nurse (RN, LPN, LVN, etc.)	Physician assistant	Pharmacist
Podiatrist	Behavioral health professionals (marriage and family therapists, medical and public health social workers, mental health and substance abuse social workers, psychologists, and mental health counselors)	Advanced practice nurses (nurse practitioners, nurse anesthetists, certified nurse midwives, and clinical nurse specialists)

Healthcare professionals (may be certified; no licensing requirement)		

Medical technologist and laboratory staff	Morgue assistant	Dental assistant
Diagnostic medical sonographer	Paramedic	Pharmacy technician
Medical records librarian	Biomedical engineer	Nursing assistant, tech
Chaplain	Respiratory therapist	Phlebotomist
Alternative medical practitioners (may be licensed in some jurisdictions)	Radiologic technologists and technicians	Athletic Trainer

*RN*, registered nurse; *LVN*, licensed vocational nurse; *LPN*, licensed practical nurse.

**Table 2:** Non-clinical volunteer positions.

Possessing vital skills		
Communication	Facility maintenance/construction	Transportation
Food services	Local access/political connections	Translation
Security	Waste disposal	Engineering
Computer operations/maintenance		

Other volunteers		

Runner	Transporter	Assistants to skilled personnel
Patients’ families, who routinely provide nontechnical bedside nursing care in many countries and cultures, especially on pediatric units; this care should be encouraged and expanded with non-COVID-19 patients when nursing care is limited.		
